# Agreement between referral information and discharge diagnoses according to Norwegian elective treatment guidelines – a cross-sectional study

**DOI:** 10.1186/s12913-014-0493-5

**Published:** 2014-10-29

**Authors:** Lise Lund Håheim, Jon Helgeland

**Affiliations:** Norwegian Knowledge Centre for the Health Service, PO Box 7004, St.Olavs plass, Oslo, N-0130 Norway

**Keywords:** Validity, Discharge, Diagnosis, Elective treatment, Specialist health service, Sensitivity, Referral

## Abstract

**Background:**

Norway introduced 32 priority guidelines for elective health treatment in the specialist health service in the period 2008–9. The guidelines were intended to reduce large differences in waiting times among hospitals, streamline referrals and ensure that patients accessed the necessary healthcare to which they were entitled for certain conditions. Referral information guided the priorities. As the referral information was key to future evaluation of the guidelines, this study validates the referral information in hospital patient records against discharge diagnoses, because only the discharge diagnosis is recorded in the Norwegian Patient Register (NPR) database, which is used in the main evaluation.

**Methods:**

Of the specific conditions from 10 priority guidelines, 20 were selected for review for the period 2008–9 at 4 hospitals in Norway. The ICD-10 diagnoses per disease or condition were given in retrospect by clinicians who participated in the expert groups developing the priority guidelines. Reasons for deviations between referral information and discharge diagnoses were coded into four categories, according to the degree of precision of the former compared with the latter.

**Results:**

In all, 1854 medical records were available for review. The diagnostic precision of the referrals differed significantly between hospitals, and across the 2 years 2008 and 2009. The overall sensitivity was 0.93 (95% confidence interval 0.92–0.94). For the separate conditions, sensitivity was in the range 0.60–1.00. Experience showed that it was necessary to pay careful attention to the selection of ICD-10 diagnoses for identifying patients. The medical records of psychiatry patients were unavailable in some cases and for certain conditions some were unavailable after use of our record extraction algorithm.

**Conclusion:**

The sensitivity of the referral information on diagnosis or condition was high compared with the discharge diagnosis for the 20 selected conditions from the 10 priority guidelines. Although the review assessed a limited number of the total, we consider the results sufficiently representative and, hence, they will allow use of the NPR data for analyses of the introduction and follow-up of the 32 priority guidelines.

## Background

Hospital and administrative databases in the health service are a source of valuable information for both analyses of health service quality assessment and research. Use of these data may require analyses of data validity to be made to assess variability and the degree of error in the data [1–13].

The Norwegian Directorate for Health directed the development of the priority guidelines for elective treatment in 32 distinct medical fields within the specialist health service. The aims of the guidelines were to reduce the large differences in waiting times among Norwegian hospitals, to streamline referral and to ensure patients’ legal rights to treatment.

The development of the guidelines, which specified any treatment priorities, were carried out by separate expert groups for each medical field, including clinicians, specialized practitioners, general practitioners (GPs) and representatives from particular patient organizations, as relevant [14]. The evaluation of the introduction of the 32 priority guidelines will be carried out on administrative data from the NPR which receives discharge diagnoses (ICD-10 codes) and procedures for all admissions to public hospitals, but not referral information [15,16]. However, the priority with regard to elective health treatment for the different diseases was based on conditions or diseases specified by the diagnostic code or symptom descriptions by the GP’s or medical specialist’s referral. The referral is at the discretion of the referring physician or specialist, or by internal referral, as per a letter or internal note/electronic message in hospitals. The hospital must respond within 2 weeks and, if the referral is about a condition that should receive priority treatment, the patient should receive a fixed date for treatment or examination within the timeframe given in the relevant priority guideline.

It is interesting to use hospital or administrative databases for epidemiological research or quality projects in the public health service. Some comparisons, a few referred to here, have been made comparing administrative databases with clinical records [1–13]. Norwegian studies include validation of a Norwegian stroke register, including non-hospitalized and hospitalized cases, to discharge diagnosis; they found a sensitivity of 0.86 for ICD-9 codes 430–438 of cerebrovascular disease [4]. Only 4.6% of the discharge diagnoses were classified as non-stroke ones. Furthermore, they found that the distinction between subtypes should not be made unless coding practices were improved. Thomsen et al. were satisfied with the validity of the diagnosis of pre-eclampsia over the period 1967–2005 in the Medical Birth Registry of Norway [12]. A single hospital evaluation of hip replacements during 1999–2002, for the NPR, and 1987–2003, for the Norwegian Arthroplasty Register (NAR), found that the NPR missed 3.4% and the NAR missed 0.4%, confirming the latter database to be valid and reliable [13]. This contrasts with the report in 2005 by Lofthus et al. which questioned the validity of electronic databases for the registration of hip fractures [11]. The total number of hip fractures, confirmed by review of medical records and logbooks of operating theatres, showed that the NPR database had over-estimated the number of fractures by 19%, whereas local electronic databases had both over- and under-estimated [11]. Since these publications, the NPR established a database in 2007 using person-identified records. The ultimate validity in fatal events comes from postmortem examinations, which Gulsvik et al. used to assess the validity of the mortality statistics for fatal cerebral stroke and coronary deaths in Bergen, Norway [3]. For fatal stroke they found a sensitivity of 0.75 (95% confidence interval [CI] 0.66–0.83) and a positive predictive value of 0.86 (CI 0.77–0.92). For coronary deaths, the values were 0.87 (CI 0.84–0.91) and 0.85 (CI 0.81–0.89), respectively.

Sørensen et al. have developed a framework for evaluating secondary data sources for epidemiological research [17]. They have outlined seven criteria to be fulfilled before administrative data can be used in research:

1. Completeness of registration of individuals
2. Accuracy and degree of completeness of the registered data
3. Size of the data source
4. Registration period
5. Data accessibility, availability and cost
6. Data format
7. Possibilities of linkage with other data sources (record linkage).

Thus, by using the NPR database we address the second criterion of accuracy and degree of completeness of the registered data. In the current study the sensitivity is an estimate of the agreement between the referral information and the hospital data [18].

The aim of the current study was to compare the accuracy and degree of completeness of the referral information with the registered discharge diagnoses by reviewing patient records of 20 selected diagnoses and conditions from 10 priority guidelines in the period 2008–9, when the priority guidelines were being implemented.

## Methods

Although 32 priority guidelines had been implemented, only 10 were found to be feasible for a study estimating the validity of discharge diagnoses recorded in the NPR (Table 1). The selection was the result of the selection criteria, sample size calculation and resources needed for this study. The NPR was established in 1997 and is a register of all people awaiting or receiving treatment in the Norwegian specialist health service. The purpose of the register is fourfold:

1. Provide data for administration, policy and quality of the specialist health service including activity-based financing.
2. Contribute to medical research including research on the health services, effect of treatment, diagnoses and disease causes, prevalence, progress and preventive measures.
3. Be the foundation for the establishment and quality assessment of hospital and quality registers.
4. Contribute to knowledge about safety and accident prevention.

**Table 1:** **Priority guidelines, diagnoses/conditions and the ICD-10 codes for the validation study**

**Priority guideline**	**Selected diagnosis/health condition**	**ICD-10 code**
Oncology	Morbidity after cancer treatment	Z08
	Brain metastases	C79.3
Oral surgery and oral medicine	Facial pain	G50, G51
	Jaw infections	K10, K04
Geriatrics	Sequel after stroke	I69.1, I69.3, I69.4
	Complex cognitive reduction	F00, F01, F03, F04, F05, F06.7, R41.8
Women’s diseases	Ovarian cancer	C56
	Descensus uteri	N81
Urology	Haematuria	N02, R31, Z03.1, Z03.8
	Kidney or ureter stone	N20, N22, N23
Lung disorders	Chronic obstructive pulmonary disease (stages 3 and 4)	J44, J44.0, J44.1, J44.8, J44.9
	Sleep apnoea	G47.3
Heart disease	Coronary heart disease (angina pectoris)	I20, I25
	Heart valve disorder	I27, I28, I34, I35, I36, I37, I38, I39
Skin disorders	Leg and foot wounds	L97
	Psoriasis: serious or moderate	L40
Mental health of children and young people	Eating disorder	F50
	Serious concern of mental disorder and behavioural disorder	Z13.3
Orthopaedics	Metastasis to muscles and skeleton	C79.5
	Hip osteoarthritis	M16

Since 2008, the registry has provided patient identifiable data.

The Norwegian Knowledge Centre for the Health Service (Knowledge Centre) initially used a two-stage process to select conditions in the relevant medical fields. A team of three researchers independently suggested five guidelines, each of which gave a broad representation of the medical fields in the guidelines. This was a challenge because there were 32 guidelines with a total of 399 conditions; they were reduced to 11 guidelines. The criteria for selection were medical field, age (adult versus children), gender-specific disorders, volume of hospital discharges per relevant diagnosis and disease severity. From each of the 11 guidelines 2 conditions or diseases were chosen in view of the following criteria: the grade of priority for elective treatment, urgency of treatment, chronic disease versus non-elective treatment and volume/prevalence (Table 1).

The estimated sample size required for the study was calculated as 92 records per selected condition, giving 95% confidence intervals with an expected half-width of 10%. This allowed assessment of 22 conditions in 11 guidelines, within a frame of 2000 records, for review. A pilot study reduced the number of guidelines to 10 because adult psychiatry data proved difficult to access. Therefore, 100 records per selected prioritized condition in 10 guidelines were chosen. The Knowledge Centre was given permission by the Norwegian Data Directorate and the Regional Committee for Ethics in Research to examine medical records at the hospitals.

The expert groups for the different guidelines selected and defined the conditions according to clinical signs and symptoms of disease. They did not give them ICD-10 or procedure codes. To ensure the quality of the ICD-10 and procedure codes used, all the expert groups were contacted to help compile this information. The expert groups provided information in all but two cases, in which physicians at the Knowledge Centre assisted. Despite this effort, not all the 399 conditions could be identified by these codes.

Hospital records provided both referral information and discharge diagnoses, avoiding the need to search across multiple primary care systems. We did not have the opportunity to select records using the referral information in the hospital’s data systems because this was incomplete for our purposes.

The following hospitals were visited: Haukeland University Hospital in Bergen (Haukeland), University Hospital of North Norway in Tromsø (UNN), St.Olav Hospital, Trondheim University Hospital in Trondheim (St Olav) and Akershus University Hospital (AHUS), Lørenskog. These hospitals were considered to have enough patients representing each of the four health regions of Norway. The four hospitals were initially chosen to ensure that we had the greatest chance of identifying sufficient relevant cases at each hospital. A visit to all Norwegian hospitals would have been costly with a minimum benefit to the project. All hospital data are stored in electronic systems. The letters of referral were scanned and added to the relevant medical record. Receipt of the referral was noted in the system. The medical record system in use was DOCULIVE at St Olav and DIPS at Haukeland, UNN and AHUS. An initial feasibility analysis was performed at UNN in November 2010. Two people estimated the concordance of their interpretations of the extracted record information, which was found to be satisfactory. The record review was subsequently delayed for administrative reasons, restarted in May 2012 and completed in June 2013.

The hospitals were asked to extract records in line with our specifications:

1. Select patients with the listed main discharge diagnoses at the first consultation or treatment session after the referral had been received.
2. In the period 2008–9 choose one patient each month who has been referred and treated, and the twenty-fifth patient from any month. The referral date should preferably not be before 1 January 2007.
3. If there is no relevant patient in a particular month, select two the next month.
4. Select only patients referred for elective treatment, not emergency treatment.
5. The list must contain different individuals.

For certain diagnoses, there were fewer than 100 patient records available so that our target of 2000 patients was not achieved. The data were recorded on a separate PC offline and no data were transferred on the web.

The statistical analyses involved estimating sensitivity overall by hospitals and by condition. The true cases of this study have matching referral information and discharge diagnosis. The sensitivity is the proportion of cases with the given discharge diagnosis that are identified by the referral information. The need to select samples on the basis of discharge diagnosis codes excludes the estimation of the specificity of referral information. Non-identification was due to different causes such as poorly formulated referral, faulty assessment or missing information. We also regarded an inaccurate discharge ICD-10 diagnosis code, compared with the written diagnosis on the record, as non-identification. The agreement of true cases was further divided into three categories to assess precision of the referral information to identify true cases:

1. Clear agreement between referral and discharge
2. Poorly formulated referral, some modification by the hospital
3. Not sufficiently specific but adequate.

An overall comparison of precision between hospitals was done by the χ^2^ test for 2008 and 2009 separately, and combined. Age is presented by median and interquartile values. Analyses were performed using SPSS Software, version 15.0.

## Results

In all, 1854 medical records were reviewed concerning 20 conditions selected from 10 priority guidelines, to give a broad insight into hospital referrals (Table 1). They had a fairly equal sex distribution (53.9% women and 46.1% men), with two conditions that concerned just women (Table 2). The age ranged from <1 year to 108 years. The study also tested the codes from the priority guidelines and, apart from the condition of haematuria at the Haukeland University Hospital, where the Z-codes Z03.1 and Z03.8 were found be insufficiently specific for the condition, the codes identified the cases. These selection criteria were removed in the subsequent reviews at the other three hospitals (Table 3). Medical records for mental health of children and young people proved difficult to access and, for some diagnoses, hospitals did not have the required number of relevant records for review.

**Table 2:** **Number of medical records examined, gender and age (years) of patients per condition or disease**

**Condition or disease**	**Number of records**	**Gender**		**Age as median (interquartile range)**	
		**Women no. (%)**	**Men no. (%)**	**Women**	**Men**
1. Eating disorder	60	58 (97)	2 (3)	21 (19–28)	29 (17–41)
2. Serious concern of mental disorder and behavioural disorder	9	6 (67)	3 (33)	14 (11–18)	17 (10–20)
3. Sleep apnoea	100	24 (24)	76 (76)	55 (33–61)	50 (40–60)
4. Chronic obstructive pulmonary disease (stages 3 and 4)	99	53 (545)	46 (47)	69 (64–78)	71 (64–78)
5. Ovarian cancer	100	100 (100)	−	65 (55–72)	−
6. Descensus uteri	100	100 (100)	−	64 (54–73)	−
7. Kidney or ureter stone	100	50 (50)	50 (50)	54 (38–66)	60 (51–74)
8. Haematuria	100	48 (48)	52 (52)	59 (46–68)	61 (41–72)
9. Hip osteoarthritis	100	69 (69)	31 (31)	69 (60–77)	63 (56–73)
10. Metastasis to muscles and skeleton	100	39 (39)	61 (61)	63 (56–68)	61 (60–80)
11. Brain metastases	100	43 (43)	57 (57)	63 (53–73)	67 (59–73)
12. Morbidity after cancer treatment	100	44 (44)	56 (56)	59 (42–74)	64 (53–69)
13. Leg and foot wounds	100	47 (47	53 (53)	80 (71–85)	66 (53–78)
14. Psoriasis: serious or moderate	90	40 (44)	50 (56)	47 (30–60)	56 (45–62)
15. Jaw infections	95	49 (52)	46 (48)	45 (31–67)	48 (27–62)
16. Facial pain	100	60 (60)	40 (40)	51 (38–65)	53 (39–65)
17. Sequel after stroke	100	38 (38)	62 (62)	69 (56–83)	67 (54–78)
18. Complex cognitive reduction	100	47 (47)	53 (53)	68 (60–79)	67 (61–72)
19. Coronary heart disease (angina pectoris)	101	39 (39)	62 (61)	65 (48–76)	65 (58–79)
20. Heart valve disorder	100	46 (46.0)	54 (54.0)	74 (62–81)	72 (61–80)
**Total**	**1854**	**1000 (53.9)**	**854 (46.1)**	**62 (48–74)**	**63 (51–73)**

**Table 3:** **Sensitivity of the referral text or referral diagnosis versus discharge diagnosis**

**Selected condition or disease specified in guideline**	**Hospital**								**Total**		
	**Haukeland (H)**		**UNN**		**St Olav**		**AHUS**				
	**No.**	**Sensitivity**	**No.**	**Sensitivity**	**No.**	**Sensitivity**	**No.**	**Sensitivity**	**No.**	**Sensitivity**	**95% CI**
1. Eating disorder	0	−	10	1.00	25		25	1.00	60	1.00	−
2. Serious concern mental disorder and behavioural disorder	0	−	−	−	−	−	9	1.00	9	1.00	−
3. Sleep apnoea	25	1.00	25	0.96	25	1.00	25	0.96	100	0.98	0.97–0.99
4. Chronic obstructive pulmonary disease	24	0.88	25	0.92	25	0.92	25	0.92	99	0.91	0.88–0.94
5. Ovarian cancer	25	0.92	25	1.00	25	0.96	25	1.00	100	0.97	0.95–0.99
6. Descensus uteri	25	0.88	25	0.96	25	1.00	25	1.00	100	0.96	0.94–0.98
7. Kidney or ureter stone	25	0.92	25	1.00	25	1.00	25	0.96	100	0.97	0.95–0.99
8. Haematuria^a^	25	0.08	25	0.96	25	1.00	25	0.92	100	0.87	0.84–0.90
9. Hip osteoarthritis	25	0.88	25	0.96	25	0.92	25	0.68	100	0.85	0.81–0.89
10. Metastasis to muscles and skeleton	25	1.00	25	1.00	25	1.00	25	0.96	100	0.99	0.97–1.00
11. Brain metastases	25	1.00	25	0.96	25	1.00	25	1.00	100	0.99	0.97–1.00
12. Morbidity after cancer treatment	25	0.96	25	0.96	25	1.00	25	0.96	100	0.97	0.95–0.99
13. Leg and foot wounds	25	1.00	25	0.92	25	1.00	25	0.96	100	0.92	0.89–0.95
14. Psoriasis: serious or moderate	25	0.92	25	0.88	25	0.96	15	0.60	90	0.87	0.84–0.91
15. Jaw infections	25	0.96	25	0.99	25	1.00	20	0.60	95	0.88	0.85–0.91
16. Facial pain	25	0.92	25	0.96	25	1.00	25	0.96	100	0.96	0.94–0.98
17. Sequel after stroke	25	0.92	25	1.00	25	1.00	25	0.96	100	0.96	0.94–0.98
18. Complex cognitive reduction	25	0.96	25	1.00	25	0.92	25	0.88	100	0.94	0.92–0.96
19. Coronary heart disease (angina pectoris)	26	0.92	25	1.00	25	0.88	25	0.92	100	0.93	0.88–0.99
20. Heart valve disorder	25	0.76	25	0.97	25	1.00	25	1.00	100	0.94	0.92–0.96
**Total**	**425**	**0.92** ^**a**^	**460**	**0.97**	**475**	**0.96**	**469**	**0.92**	**1829** ^**a**^	**0.93**	**0.92–0.94**

^a^Haematuria at Haukeland: the two codes used did not identify the cases and the result was discarded. These codes were not used in the other hospitals. The total number of medical records reviewed was 1854, but 25 were discarded.

AHUS, Akershus University Hospital; CI, confidence interval; UNN, University Hospital of North Norway in Tromsø.

The referral information was coded to show how well it matched the discharge diagnosis (Table 4). The frequencies of these categories varied between the hospitals in both 2008 and 2009, and in combination (χ^2^ test; *p* <0.05). The sensitivity increased after adding less specific but sufficient referral information to the definite referral diagnosis and information. For Haukeland the sensitivity increased from 74.4 to 88.2 (including haematuria), for UNN from 83.9 to 96.3, for St Olav from 89.5 to 95.4 and for AHUS from 88.5 to 91.7. The reasons for incorrect referrals were poor formulation, faulty physician assessment or lack of information (*n* = 10, 0.6% of total). In the course of the record review, discharge diagnoses were identified by chance that did not have a corresponding correct referral for the treatment subsequently given (*n* = 121, 6.5% of total).

**Table 4:** **The agreement or sensitivity for referral codes or text versus discharge diagnoses**

**Definitions of agreement or lack of agreement** ^**a**^	**Hospital**				
	**Haukeland No. (%)**	**UNN** ^**a**^ **No. (%)**	**St Olav No. (%)**	**AHUS** ^**a**^ **No. (%)**	**Total No. (%)**
1. Clear agreement between referral and discharge	335 (74.4)	386 (83.9)	425 (89.5)	415 (88.5)	1561 (84.2)
2. Poorly formulated referral; some modification by the hospital	48 (10.7)	19 (4.1)	3 (0.6)	15 (3.3)	85 (4.6)
3. Not sufficiently specific but adequate	14 (3.1)	38 (8.3)	25 (5.3)	0 (0)	77 (4.2)
Good agreement: sum of codes 1–3	397 (88.2)	443 (96.3)	453 (95.4)	430 (91.7	1723 (92.9)
4. No agreement	53 (11.8)	17 (3.7)	22 (4.6)	39 (8.3)	131 (7.1)
Total number of records assessed	450 (100)	460 (100)	475 (100)	469 (100)	1854 (100)
Sensitivity^a^	0.88	0.96	0.95	0.92	0.93
95% confidence interval	0.87–0.90	0.94–0.98	0.91–0.99	0.90–0.94	0.92–0.94

^a^The codes 1–3 are valid diagnoses and code 4 is a non-valid diagnosis.

Range of values: 0–1.00; study period 2008–9. **χ** ^**2**^ tests overall 2008, 2009, 2008–9, and between hospitals 2008–9 – all *p* <0.01.

AHUS, Akershus University Hospital; UNN, University Hospital of North Norway in Tromsø.

Sensitivity was found to be very high overall and per condition or disease (see Table 3). The overall sensitivity was 0.93 (95% CI 0.92–0.94). Comparative values for the hospitals were: Haukeland 0.92 (excluding haematuria), UNN 0.96, St Olav 0.95 and AHUS 0.92. Overall, the sensitivity per condition ranged from 0.87 (hip osteoarthritis, *n* = 100) to 1.00 (eating disorder, *n* = 25). Values of sensitivity <0.80 were: hip osteoarthrosis 0.71, psoriasis – serious or moderate – 0.60 and jaw infections 0.60, all at AHUS, and heart valve disorder 0.76 at Haukeland.

## Discussion

The overall agreement between the discharge diagnoses for elective treatment and the referrals was high. The sensitivity ranged from 0.88 (0.87–0.90) at Haukeland, to 0.96 (0.94–0.98) at UNN, 0.95 (0.91–0.99) at St Olav and 0.92 (0.90–0.94) at AHUS, and overall 0.93 (0.92–0.94). The referral information was categorized according to the level of precision, and there was no common trend towards systematic bias. The level of precision differed significantly between the hospitals in 2008 and 2009, and combined. Although the precision in the referrals differed, the high degree of sensitivity indicated the high accuracy of the information re the conditions and other relevant information in the referrals, both external and internal. The correct coding of the prioritized diseases or conditions was critical for identifying the patients, as shown with haematuria. The main categories of the ICD-10 are more precise and subsidiary diagnoses should be used in combination. The age ranged from <1 year to 108 years, and males and females were reasonably represented, giving confidence in the representative nature of the samples taken from four major hospitals in each of the four regional health regions of Norway.

The Norwegian studies mentioned earlier [3,4,11-13] and international studies confirm a degree of variability in sensitivity, specificity and positive predictive values [1,2,5–10]. Lacasse et al. underlined the importance of routinely assessing the validity of diagnoses before making use of administrative databases in research [9]. Validity studies from other countries include the Finnish Hospital Discharge Register, which made a systematic overview of 32 studies that had validated their data, many using medical records [1]. They assessed the completeness and accuracy as ranging from satisfactory to very good. Two validity studies of the Danish National Registry of Patients (DNRP) found, after reviewing medical records, a sensitivity of 0.91 for pulmonary empyema overall, but sensitivity decreased with patient age. Another study of the DNPR on atrial fibrillation and atrial flutter was also satisfactory, but the researchers found that the precision of the ICD-10 coding was important for correctly identifying cases [6,7]. Our results are in line with these studies. Herrett et al. compared the different sources of data from primary care, hospital care, disease registry and national mortality records in England [2]. Investigating the incidence of acute myocardial infarction, they found that use of a single source, compared with all three sources of information, on average under-estimated the incidence by 25–50%. In that study the disease registry was taken as the gold standard. The sensitivity of primary care data was 0.93 (95% CI 0.92–0.93) and for hospital admissions 0.925 (0.91–0.92).

The source of data is important, because Lee et al. found that the validity of hospital discharge data was insufficient to get the incidence for several cancer diagnoses [5]. In the UK a validation study of cancer diagnoses in the general practice research database (GPRD) and of the Cancer Registry (CR) database was carried out [10]. There were 91% cancer events in both databases. The researchers found that false-negative primary care records were due to a delay by an average of 11 days from registration in the GPRD to that in the CR database. When comparing data sources attention must be paid to disease codes and dates for recording diagnoses. Examining the validity of heart failure diagnoses, Lee et al. compared ICD-9 codes and the Framingham criteria, and in addition studied the impact of including Charlson’s index of co-morbidity [9]. The ICD-9 codes were highly predictive and co-morbidity information could enhance future studies on heart failure mortality. This aspect is relevant if the objective is to gain further understanding of single diagnoses.

The current study has contributed to increased knowledge in Norway about the validity of referral information versus discharge diagnosis. Ideally, the sample size should have been larger and all the guidelines should have been examined in this manner, ready for our main study. However, this was found not to be feasible. If the results of this validation had indicated poor agreement between referral information and discharge diagnosis, the main study would have been more limited. Errors that occurred were wrongly coded discharge diagnoses, an example being a neurological lesion in a foot coded as Bell’s palsy based on the guideline on oral surgery and oral medicine. Real changes in diagnosis from referral to discharge were few. The differences in the two electronic record systems did not represent a big problem for extracting the data. However, information in the records was sometimes missing and, in some cases, inaccessible to the reviewer. Incomplete recording occurred commonly. This study could not address the specificity of the referral information. Another of its limitations is that it relies on a single observer (LLH) abstracting records and judging agreement, although the pilot study did show agreement between two observers.

The current study does not validate NPR data as such against hospital discharge registers. In the period under study, there was a transition from CD to electronic transfer of data (in 2008 about 50:50; in 2013 only a few minor units reported by sending CDs). However, an assumption was made that the electronic transfer of data from the hospital discharge registers to the NPR functions well. The codes are quality assured before being transmitted to the NPR. The NPR in turn also performs quality controls and reports back to the hospitals, which return corrected data to the NPR. This study does, however, give an estimate of the accuracy and degree of completeness of the registered data in the hospital records and thus in the NPR. Hence, it gives an overall estimate of error in the NPR data; this can be used to identify patients within the different conditions of the 32 guidelines.

## Conclusion

In view of these results, we believe that the referral information agrees with the discharge diagnosis with a sufficiently high sensitivity among patients with the discharge diagnoses tested. The challenge is to correctly specify the codes of the conditions in the priority guidelines. A limitation of the main study will be the inability to follow those clinical conditions that did not have identified ICD-10 or procedure codes. Although this limits the number of conditions studied, we believe that the NPR databases can be used with reasonable confidence for the purpose, in our main study, of analysing the introduction of 32 priority guidelines, knowing the estimated overall level of error.
